# Access to syringes for HIV prevention for injection drug users in St. Petersburg, Russia: syringe purchase test study

**DOI:** 10.1186/1471-2458-13-183

**Published:** 2013-03-01

**Authors:** Ekaterina V Fedorova, Roman V Skochilov, Robert Heimer, Patricia Case, Leo Beletsky, Lauretta E Grau, Andrey P Kozlov, Alla V Shaboltas

**Affiliations:** 1 The Biomedical Center, 8, Viborgskaya Street, St. Petersburg 194044, Russia; 2 Saint Petersburg State University, 7-9, Universitetskaya nab, St. Petersburg 199034, Russia; 3 Center for Interdisciplinary Research on AIDS, School of Public Health, Yale University, 60 College Street, PO Box 208034, New Haven CT 06520-8034, USA; 4 The Fenway Institute, Fenway Health, 1340 Boylston Street, Boston, MA 02215, USA; 5 Northeastern University School of Law & Bouvé College of Health Sciences, 400 Huntington Ave, Boston, MA 02115, USA

**Keywords:** Injection drug use, HIV prevention, Syringe access, Pharmacy

## Abstract

**Background:**

The HIV epidemic in Russia is concentrated among injection drug users (IDUs). This is especially true for St. Petersburg where high HIV incidence persists among the city’s estimated 80,000 IDUs. Although sterile syringes are legally available, access for IDUs may be hampered. To explore the feasibility of using pharmacies to expand syringe access and provide other prevention services to IDUs, we investigated the current access to sterile syringes at the pharmacies and the correlation between pharmacy density and HIV prevalence in St. Petersburg.

**Methods:**

965 pharmacies citywide were mapped, classified by ownership type, and the association between pharmacy density and HIV prevalence at the district level was tested. We selected two districts among the 18 districts – one central and one peripheral – that represented two major types of city districts and contacted all operating pharmacies by phone to inquire if they stocked syringes and obtained details about their stock. Qualitative interviews with 26 IDUs provided data regarding syringe access in pharmacies and were used to formulate hypotheses for the pharmacy syringe purchase test wherein research staff attempted to purchase syringes in all pharmacies in the two districts.

**Results:**

No correlation was found between the density of pharmacies and HIV prevalence at the district level. Of 108 operating pharmacies, 38 (35%) did not sell syringes of the types used by IDUs; of these, half stocked but refused to sell syringes to research staff, and the other half did not stock syringes at all. Overall 70 (65%) of the pharmacies did sell syringes; of these, 49 pharmacies sold single syringes without any restrictions and 21 offered packages of ten.

**Conclusions:**

Trainings for pharmacists need to be conducted to reduce negative attitudes towards IDUs and increase pharmacists’ willingness to sell syringes. At a structural level, access to safe injection supplies for IDUs could be increased by including syringes in the federal list of mandatory medical products sold by pharmacies.

## Background

The HIV epidemic in Russia, which began in the late 1990’s, has been largely concentrated among people who inject drugs [1-3]. Through 2009, 567,558 cases of HIV have been identified, with more than 10% added in 2009 alone [4]. Estimates suggest that more than 1.2 million Russians are infected with HIV, with 82% reporting injection drug use (IDU) [1,5].

St. Petersburg, the second largest city in Russia with a population of 5 million registered inhabitants, experienced an expanding heroin market in the late 1990’s and a subsequent epidemic of HIV. Presently, among the city’s estimated 83,000 IDUs [6], about 82% of IDUs inject heroin as their primary drug, and about 18% inject ephedrine-based stimulants [7]. As of 2009, more than 38,000 individuals had been diagnosed with HIV with 76.4% of cases attributed to injection drug use [8]. HIV prevalence among St. Petersburg IDUs is alarmingly high, increasing from <5% prior to 2000 to 30% in 2003 and to 44% in 2007 [9-11]. In longitudinal cohorts of IDUs, HIV incidence was 4.5 per 100 person-years in 2003 and 7.2 per 100 person-years in 2009 [12,13].

HIV transmission among IDUs results primarily from unsafe injection practices such as the sharing of used syringes due to the lack of access to sufficient supply of sterile syringes [14,15]. This is often considered a structural issue related to law and public policies. This has been especially true in the U.S., where laws have restricted access [16-18]. Outside of the U.S. where access is not legally restricted, the extent to which local pharmacies are willing to sell syringes to IDUs is an important factor shaping epidemic control efforts. In Edinburgh, Scotland, for example, an outbreak of HIV among IDUs in the mid-1980’s has been attributed to a decision reached by pharmacists and the police to halt sales of syringes to customers who appeared to be IDUs by subjective judgment [19]. Studies on expanding access have been conducted in several locations, most notably in the U.S. [20,21], in the wake of changing laws that allowed purchase and possession of syringes without prescription [22,23]. Large-scale expansion of targeted sales of syringes to IDUs from pharmacies has been shown to contribute to maintaining low prevalence of HIV-1 among Australian and British IDUs and lowering HIV incidence in New York City [24-26]. However, most studies on syringe access, even those undertaken most recently [27,28], have focused on locations in which syringe sales had been legally restricted in some way. The situation is very different in St. Petersburg, Russia.

According to Russian federal laws and regulations, syringes can be sold in all pharmacies without legal limitations. However, there are often gaps between the formal laws and their street-level implementation [16,29,30]. Syringes and condoms are not included in the required pharmacy formulary as stated in the governing decree, “On minimal assortment of medicinal agents” and approved by the Ministry of Healthcare and Social Development [31]. Accordingly, availability of syringes in a pharmacy is not regulated by any legal act but varies with commercial polices of the specific pharmacies. Thus, declining to sell syringes is not a violation. It is important to note that since there are very few syringe exchange programs in St. Petersburg, pharmacies are the primary source of syringes for IDUs [32].

The “International Feasibility Study of Pharmacy-Based HIV Prevention” is a multi-site, cross-sectional, multi-phase, multi-methods research project – three sites are located in the U. S. and the other three – in China, Russia and Vietnam. The aim of the collaborative project is to evaluate the feasibility of implementing pharmacy-based interventions to reduce HIV transmission among IDUs. In this manuscript, we examine syringe access at the pharmacies of St. Petersburg including stocking and sales practices as one of the multi-site study objectives. Specifically, we explored the role of syringe access ecologically and, focusing on two of the city’s 18 districts, examined how pharmacies’ business models and their stocking and sales practices may influence access. First, we tested the hypothesis that since syringe access was not legally restricted, those districts with a higher pharmacy density would have lower rates of HIV infection. Second, we sought to understand differences between policy and practice by attempting to purchase syringes at each pharmacy in the two districts.

## Methods

To evaluate syringe access in St. Petersburg, pharmacy mapping, a telephone survey of pharmacists, qualitative interviews about the actual experiences of active IDUs with pharmacists, and a syringe purchase test were conducted from November 2009 through June 2010.

### Citywide pharmacy mapping

First, we identified and mapped pharmacies throughout the city to evaluate whether pharmacy density could explain the epidemic pattern at the district level. We used the Russian search term “аптека” [pharmacy] on Google maps to identify pharmacy location and Quantum GIS to generate a map of pharmacy density in relationship with data on HIV prevalence in residential areas of the city [33]. We obtained the official data on HIV prevalence by district from the City AIDS Center and data on population density by district from the official electronic source of the Federal Statistics Service [8,34]. Pearson correlation coefficient was used to test the hypothesis that the population-adjusted pharmacy density and HIV prevalence were statistically correlated.

### Selection of two study districts

Given the large number of pharmacies citywide, we decided to select two city districts that differed based on (1) proximity to the city center and (2) the distribution of commercial to residential space. The Petrogradskiy district is located in the city center, and encompasses 24 square kilometers containing commercial, recreational, and residential facilities. As of 2009, its population was 124,800 inhabitants (population density: 5,200 persons per square kilometer); the cumulative number of registered drug users was 138.16 per 100,000 inhabitants with HIV prevalence in the district at 586.6 per 100,000. The Viborgskiy district is located on the northern periphery of the city, and encompasses 115.4 square kilometers that is primarily residential. As of 2009, its population was 410,000 inhabitants (population density: 3,565 persons per square kilometer); the cumulative number of registered drug users was 84.81 per 100,000 inhabitants with HIV prevalence in the district at 597.86 per 100,000. Overall across the city districts, HIV prevalence varied from 339.2 to 1143.6 per 100,000, and range for the cumulative number of registered drug users was 24.53-220.42 per 100.000 inhabitants [8,34].

### Pharmacy telephone survey

We conducted a brief telephone survey at each of the pharmacies in the two selected city districts – Petrogradskiy and Viborgskiy. A staff member called each pharmacy and asked if the pharmacy stocked 1 or 2 ml syringes for sale. Although syringes are sold with volumes from 1 to 20 ml capacity, the majority of IDUs use 1 or 2 ml syringes. Data were recorded in an Excel spreadsheet.

### Qualitative interviews with IDUs

In preparation for a pharmacy syringe purchase test, we used data from 26 semi-structured interviews with a convenience sample of active IDUs. Participants were recruited from an existing cohort followed at the Biomedical Center. All participants reported injecting illicit drugs within a month prior to the interview. An interview guide was developed for this study and focused on IDUs’ past experiences with pharmacies and pharmacy staff and attitudes towards a range of possible pharmacy-based HIV prevention interventions. For this manuscript we restricted the qualitative analysis to the evaluation of IDUs’ past experiences of syringe purchase in pharmacies. Interviews were audio-recorded, transcribed, and coded using open and axial codes in a thematic analysis. The interview guide and informed consent forms were approved by the Yale University and the Biomedical Center Institutional Review Boards. Informed consent was obtained from all participants, including consent to be audio-recorded.

### Pharmacy syringe purchase test

The pharmacy syringe purchase test was conducted in all 108 pharmacies located in the two selected city districts. We developed a standard script using methods from Compton et al. [21]. The script was adapted to the Russian setting, taking into account specific injection behaviors of local IDUs.

Three Russian male research assistants, experienced in field work with IDUs, were involved in the syringe purchase test. They were trained to follow the script using the following protocol. Two members of the research staff entered the pharmacy and asked for a single 1 ml syringe and Naphazoline, a saline solution sold in glass bottles which provides IDUs with both liquid to dissolve drugs for injections and a receptacle (“cooker”) used to prepare the injection. Qualitative data indicated that these two items are the most frequently requested supplies that IDUs seek to buy. The research staff were instructed to avoid any suggestion of deception in research. There were no direct or indirect attempts, either through dress or behavioral cues or through verbal statements to suggest that the research staff were IDUs.

The standardized purchasing protocol had a number of steps. If told that a single 1 ml syringe was not available, the researcher was instructed to ask for a single 2 ml syringe. If told that the pharmacy only sold syringes in packs of ten, the research staff asked for and purchased a pack of ten. If informed that the pharmacy sells syringes only in quantities greater than ten, the research staff inquired about the minimum quantity of purchase and then declined to purchase. If informed that the pharmacy did not sell 1 or 2 ml syringes, the research staff would ask the pharmacist whether other kinds of syringes were for sale, and then decline to purchase any alternative syringes that were available. All purchases of 1 or 2 ml syringes either as single syringes or in packs of ten were counted as a successful purchase.

A debriefing form was completed after each pharmacy syringe purchase test that included information about the type, number and price of syringes purchased, and, if the purchase was unsuccessful, research staff provided additional comments. Statistical analysis was performed with SAS software (version 9.1, Cary, NC). Fisher’s Exact Test was used to explore associations of pharmacy types with measures of syringe access.

## Results

### Citywide pharmacy mapping

A total of 965 pharmacies were identified citywide (see Additional file 1). All were retail pharmacies and could be divided into three types: chain pharmacies (n = 694), independent pharmacies (n = 189) that included veterinary, homeopathic and clinic-based pharmacies, and pharmacies subsidized through a governmental program (n = 82) to provide certain citizens with low-priced medical supplies. No significant statistical associations were found between HIV prevalence and pharmacy density on the district level regardless of whether pharmacy density was measured per square kilometer or per 100,000 inhabitants (p = 0.61 and p = 0.24, respectively) (see Additional file 2).

### Pharmacy telephone survey

The telephone survey was conducted with the 108 pharmacies identified in the two selected city districts: 89 (82%) reported having 1 or 2 ml syringes available for sale, and 19 (18%) reported stocking neither type. However, 19 pharmacies that reported stocking 1 or 2 ml syringes over the phone survey later declined to sell them in the purchasing test, claiming not to stock them.

### Qualitative interviews with IDUs

The syringe purchase test script was developed on the results of the qualitative interviews with a convenience sample of 26 IDUs (18 males and 8 females) who reported injecting within 30 days prior to the interview. Three of the eight females were sex workers. Twenty-four respondents reported heroin as their drug of choice, one respondent reported methadone, and one respondent reported homemade ephedrine-based stimulants.

Twenty respondents believed that pharmacists could distinguish IDUs from other customers either by their appearance (n = 14) or based on the purchase of a glass bottle of Naphazoline and 1 or 2 ml syringe (n = 6), which was reported as a standard purchase made by IDU as was mentioned by 13 interviewees.

“Pharmacists don’t sell insulin syringes and Naphazoline because they know that junkies use Naphazoline as a cooker for heroin.” (Heroin user, female, 26 years old)

Difficulties in purchasing 1 or 2 ml syringes were reported by 23 interviewees. Four noted that pharmacists openly asked them to leave the pharmacy and in two of those instances threatened to call the police. One participant described being asked to leave the pharmacy repeatedly by one specific pharmacist every time he tried to purchase syringes.

“Pharmacists who are normal persons just tell you that they don’t have syringes. In other words, they talk to you in this way if they don’t want to be rude. However, most of the time they just…told me to get away twice saying – “If you start to argue we will call the police.” (Heroin user, male, 30 years old)

“Some [pharmacists] are just telling you without shame: “We have nothing – no insulin syringes, no Naphazoline – go away from here!” (Heroin user, male, 35 years old)

“Another pharmacist does sell, but this one [in the same pharmacy] doesn’t have syringes any time we come. She is always saying something like – “I have nothing… get away from here… you are junkies.” (Heroin user, male, 34 years old)

Four respondents reported witnessing pharmacists selling syringes to other customers who respondents believed were non-IDU customers while their own requests to purchase syringes were denied by the pharmacists.

“They look at you like you are a public enemy and declare that they don’t have any syringes in stock. Right afterwards some old lady comes over the counter and purchases syringes easily.” (Heroin user, female, 37 years old).

Six interviewees were denied purchase of a single syringe and were offered a 10- or 50-syringe pack instead. Four IDUs believed that pharmacists employed this practice to discourage IDUs from going to their pharmacy in order not to have IDUs scare off other customers.

“Maybe they are trying to deter drug addicts [from coming to a pharmacy]…in other words they have a somewhat negative attitude to the people who use drugs who are considered as “garbage” of society.” (Heroin user, male, 33 years old)

“Junkies would scare off “normal public” who would try to avoid making purchases in this pharmacy.” (Heroin user, female, 37 years old).

### Pharmacy syringe purchase test

Among 127 mapped pharmacies in two city districts, 108 were included in the syringe purchase test; 19 pharmacies were not operating at the time of the study. Overall, 70 (65%) of purchase attempts resulted in successful purchase of 1 or 2 ml syringes: 49 pharmacies sold as singles and 21 in packs of ten. When syringes were sold, the price ranged from 2 to 10 rubles (USD $0.07-$0.30) for a single syringe and 42 to 59 rubles (USD $1.40-$2.00) for packs of ten.

### Petrogradskiy district

Five of the 29 mapped retail pharmacies were not located. The final list of 24 pharmacies included one pharmacy involved in the governmental subsidy program, one independent pharmacy, and 22 chain pharmacies. Research staff were able to purchase syringes in 11 (46%) pharmacies: nine (38%) pharmacies sold single syringes and two (8%) pharmacies sold packs of ten (Table 1).

**Table 1:** Syringe sales at pharmacies in two districts, St. Petersburg, Russia

	**Petrogradskiy**	**Viborgskiy**	P value*
Number of Pharmacies	24	84	
Refused to sell (total)	13 (54%)	25 (30%)	0.03
Stocked but refused to sell	5 (21%)	14 (17%)	0.76
Don’t stock at all	4 (17%)	7 (8%)	0.25
Don’t stock right now	4 (17%)	4 (5%)	0.07
Sold 1 or 2 ml syringes (total)	11 (46%)	59 (70%)	0.03
As singles	9 (38%)	40 (48%)	0.48
As 10 packs only	2 (8%)	19 (23%)	0.15
Pharmacy type selling syringes			
Subsidized through governmental program	0 of 1	7 of 8	0.22
Chain	10 of 22	44 of 63	0.07
Independent	1 of 1	8 of 13	1.00

* P value obtained from Fisher’s Exact Test.

We could not purchase syringes in 13 (54%) pharmacies (one pharmacy involved in the governmental subsidy program and 12 chain pharmacies); of these, five refused to sell to our research staff although it had been reported during the phone survey the day before that they had such syringes in stock.

### Viborgskiy district

Fourteen of the 98 mapped retail pharmacies, were not located. The final list of 84 pharmacies included eight pharmacies involved in the governmental subsidy program, 13 independent pharmacies, and 63 chain pharmacies. Research staff were able to buy syringes in 59 pharmacies (70%): 40 (48%) as single syringes and 19 (22%) as packs of ten (Table 1).

At 25 (30%) pharmacies (19 chain, one involved in the governmental subsidy program, and five independent) research staff could not purchase syringes; of these, 14 reported stocking 1 or 2 ml syringes during the telephone survey the day before but refused to sell to our staff.

Overall, purchasers were almost three times more likely to successfully buy syringes in the Viborgskiy district compared to the Petrogradskiy district (OR, 2.79; 95% CI, 1.10 – 7.06, p = 0.032).

## Discussion

The data presented in this manuscript constitute the first part of a project designed to assess the feasibility of conducting HIV prevention or treatment interventions through pharmacies in St. Petersburg. This phase of the study focused on determining access to syringes, a major component in HIV prevention for IDUs, by providing simple descriptive evidence of the density of pharmacies in the city of St. Petersburg and documenting the extent to which pharmacies in two districts provide syringe access to IDUs. First, we found no evidence to support the hypothesis that pharmacy density may have played a role in the HIV epidemic among IDUs, finding no association of density with HIV prevalence. This suggests that the presence of pharmacies alone may not guarantee sufficient access to syringes for IDUs to accomplish a reduction in injection risks or prevent HIV transmission. We then explored if this was due to pharmacists’ unwillingness to sell syringes to IDUs and found that, in two districts, approximately two-thirds of the pharmacies sold the types of syringes preferred by IDUs either as singles or in packs of ten. Even though the ability to purchase a pack of ten was considered as successful, it still could be considered a barrier to immediate syringe access since its price is considerably higher than for a single syringe. Furthermore, carrying a pack of syringes may be more noticeable and consequently increase risk of police harassment. Although the proportion differed by district, the data suggest that pharmacies can play in important role in HIV prevention efforts by expanding IDUs’ access to sterile syringes. Nevertheless, the qualitative data revealed that many barriers to expanded syringe access remain. Additional research is required to understand and account for the threefold difference in successful purchases between the two study districts.

Our findings parallel a previous study conducted in 2003 in three Russian cities – Moscow, Volgograd, and Barnaul – in which pharmacies were determined to be the predominant source of syringe supply for IDUs, providing approximately one syringe per day to the IDUs interviewed in these cities [15]. However, the prevailing attitudes and perceptions of pharmacists and IDUs that may influence syringe access remain unclear in different cities across the Russian Federation, and additional research is needed to clarify this issue.

Our results demonstrate that even in locations in which no laws or regulations restrict access to syringes for IDUs, access may still be limited, in part, by pharmacists’ decisions not to stock the types of syringes favored by IDUs or to refuse to sell syringes to customers perceived to be IDUs. These practices are similar to those observed in pharmacies in other countries [35,36]. Interventions aimed at improving pharmacists’ attitudes about serving IDU customers warrant development and evaluation.

In this context, pharmacies may be a promising venue for HIV prevention because of the existing laws/regulations and the density of pharmacies in major cities. Evaluating whether pharmacy networks may be used to expand existing or implement new and comprehensive prevention services for IDUs is important in developing interventions. In this phase of the study we did not collect data from pharmacists who are and are not willing to sell syringes to IDUs to determine: (1) if the reluctance of the latter group can be overcome and (2) what incentives might encourage pharmacists to expand syringe access to and provide other services for IDUs. These efforts are currently under way in the next phase of our project that includes discussions with pharmacists and public health and safety officials.

Several limitations to this study must be noted. First, it is limited to St. Petersburg, and the findings may not be generalized to other locations. Second, the lack of correlation between pharmacy density and HIV prevalence could have been influenced by other social and behavioral factors (e.g., syringe sharing, police activities) which were not assessed as part of this study. Third, according to the preliminary data analysis of the qualitative interviews, the primary factor IDUs mentioned as a reason for unsuccessful syringe purchases was the pharmacists’ negative attitude towards IDUs. Further analysis is needed to assess other factors such as gender and the number of years injecting drugs that may elicit differential discrimination. The data analysis presented in this manuscript was focused on the developing the syringe purchase test script. Fourth, attempts to purchase syringes as part of this study were not made by actual IDUs, and the success rate noted herein may represent an overestimate. It is possible that IDUs who attempt to purchase syringes may encounter greater difficulty in purchasing syringes based on their behaviors or physical appearance. Finally, all of the purchases were made by male Russian research staff between the ages of 31 and 40 years old. Individual characteristics that may influence the purchase transaction such as gender, ethnicity or age were not tested.

## Conclusions

The description of the current situation regarding access to HIV prevention materials through pharmacies is the first phase in a multi-phase process aiming to develop and implement effective interventions to improve the health of IDUs. This study is the first effort in Russia to apply a scientific approach of combining epidemiological, behavioral, qualitative, social, and geographical data to the process of developing IDU-focused HIV prevention programs. The results of the present study reveal that, despite the absence of legal limitations on availability of syringes, about one-third of pharmacies refused to sell syringes. Among the potential interventions that may improve access to syringes and reduce HIV among IDUs in St. Petersburg would be a policy change to include syringes in the federal list of mandatory medicinal products that would require pharmacies to stock syringes and interventions aimed at improving pharmacists’ attitudes about selling syringes and reducing the stigmatization of IDUs.

## Abbreviations

IDU: Injection drug users.

## Competing interest

All authors declare to have no competing interest.

## Authors’ contributions

EVF contributed to the study design, collected, analyzed the qualitative data and wrote the first draft of the manuscript. RVS contributed to the design of the study, conducted data collection, mapping and statistical analysis. RH participated in the development of the study design and research protocol, and the organization and writing of the manuscript. PC contributed to the design of the study. AVS participated in the development of the study design and research protocol, and the overall management of the project in St. Petersburg. EVF, RVS, RH, PC, LB, LEG, APK and AVS contributed to the analysis, participated in drafting of the manuscript, and critically reviewed the final draft of the manuscript. All authors read and approved the final version of the manuscript.

## Pre-publication history

The pre-publication history for this paper can be accessed here:

http://www.biomedcentral.com/1471-2458/13/183/prepub

## Supplementary Material

Additional file 1 **Distribution of pharmacies by type and HIV prevalence by districts.** Displays distribution of pharmacies by type and shows HIV prevalence by district level (St. Petersburg, Russia; 2009). Three pharmacy types are presented: government-supported, chain and independent. Data on HIV prevalence (measured per 100,000 inhabitants) is divided in 10 categories. Click here for file

Additional file 2 **Data on HIV prevalence and pharmacies’ density by district.** Displays data on HIV prevalence and pharmacies’ density in each of 18 city’s districts. Pharmacies’ density is measured per square kilometer and per 100,000 inhabitants. Click here for file
