# Supplementary material for: Access to syringes for HIV prevention for injection drug users in St. Petersburg, Russia: syringe purchase test study
Source: BMC Public Health. 2013 Mar 1;13:183. doi: 10.1186/1471-2458-13-183 (PMC3616994; doi:10.1186/1471-2458-13-183)
Supplement: Additional file 2 — Data on HIV prevalence and pharmacies’ density by district. Displays data on HIV prevalence and pharmacies’ density in each of 18 city’s districts. Pharmacies’ density is measured per square kilometer and per 100,000 inhabitants. [file 1471-2458-13-183-S2.doc]

Data on HIV prevalence and pharmacies’ density by city district

| District | HIV prevalence per 100,000 inhabitants | Area in square kilometers | Population (thousands) | N of pharmacies | Pharmacies per square kilometer | Pharmacies per 100,000 inhabitants |
| --- | --- | --- | --- | --- | --- | --- |
| Admiralteyskiy | 696.5 | 13.8 | 170.3 | 58 | 4.20 | 34.06 |
| Vasileostrovskiy | 505.3 | 14.6 | 195.1 | 51 | 3.48 | 26.14 |
| Vyborgskiy | 597.8 | 115.1 | 410.3 | 98 | 0.85 | 23.88 |
| Kalininskiy | 590.0 | 40.1 | 457 | 85 | 2.12 | 18.6 |
| Kirovskiy | 762.3 | 48.0 | 320 | 80 | 1.67 | 25 |
| Kolpinskiy | 1143.6 | 105.7 | 183.6 | 33 | 0.31 | 17.97 |
| Krasnogvadreyskiy | 828.2 | 56.8 | 323.6 | 72 | 1.27 | 22.25 |
| Krasnoselskiy | 888.2 | 115.0 | 307.8 | 48 | 0.42 | 15.6 |
| Kronshtadt | 614.3 | 15.8 | 42.7 | 17 | 1.07 | 39.8 |
| Kurortniy | 339.2 | 267.9 | 68 | 14 | 0.05 | 20.6 |
| Moskovskiy | 545.6 | 71.1 | 290.3 | 77 | 1.08 | 26.5 |
| Nevskiy | 790.0 | 61.8 | 439.8 | 106 | 1.72 | 24.1 |
| Petrogradskiy | 586.6 | 24.0 | 124.8 | 29 | 1.21 | 23.24 |
| Petrodvorcoviy | 1043.1 | 115.3 | 116.9 | 15 | 0.13 | 12.83 |
| Primorskiy | 437.5 | 109.9 | 415.8 | 128 | 1.17 | 30.78 |
| Pushkinskiy | 810.5 | 240.0 | 124.8 | 31 | 0.13 | 24.8 |
| Frunzenskiy | 581.6 | 37.5 | 391 | 71 | 1.90 | 18.16 |
| Centralniy | 811.9 | 17.1 | 218.5 | 88 | 5.14 | 40.27 |
